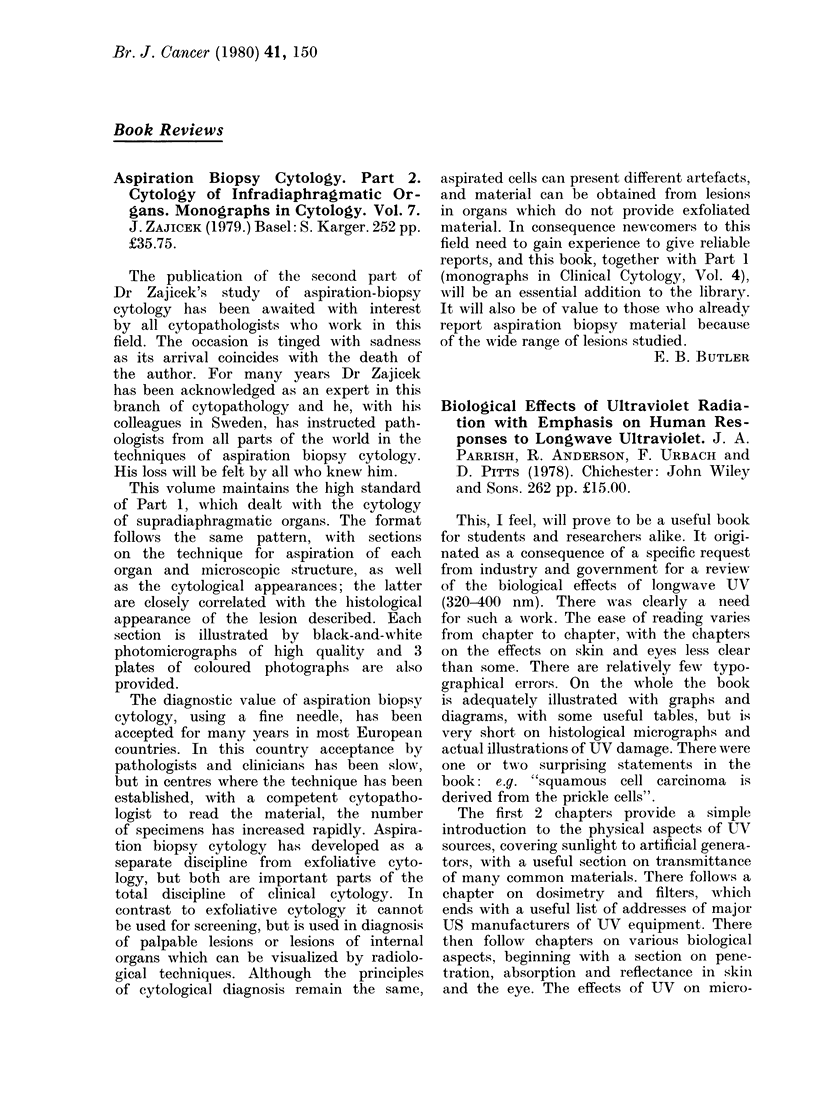# Aspiration Biopsy Cytology. Part 2. Cytology of Infradiaphragmatic Organs. Monographs in Cytology. Vol. 7

**Published:** 1980-01

**Authors:** E. B. Butler


*Br. J. Cancer* (1980) **41**, 150

### *Book Reviews*

**Aspiration Biopsy Cytology. Part 2. Cytology of Infradiaphragmatic Organs. Monographs in Cytology. Vol. 7.** J. Zajicek (1979.) Basel: S. Karger. 252 pp. £35.75.

The publication of the second part of Dr Zajicek's study of aspiration-biopsy cytology has been awaited with interest by all cytopathologists who work in this field. The occasion is tinged with sadness as its arrival coincides with the death of the author. For many years Dr Zajicek has been acknowledged as an expert in this branch of cytopathology and he, with his colleagues in Sweden, has instructed pathologists from all parts of the world in the techniques of aspiration biopsy cytology. His loss will be felt by all who knew him.

This volume maintains the high standard of Part 1, which dealt with the cytology of supradiaphragmatic organs. The format follows the same pattern, with sections on the technique for aspiration of each organ and microscopic structure, as well as the cytological appearances; the latter are closely correlated with the histological appearance of the lesion described. Each section is illustrated by black-and-white photomicrographs of high quality and 3 plates of coloured photographs are also provided.

The diagnostic value of aspiration biopsy cytology, using a fine needle, has been accepted for many years in most European countries. In this country acceptance by pathologists and clinicians has been slow, but in centres where the technique has been established, with a competent cytopathologist to read the material, the number of specimens has increased rapidly. Aspiration biopsy cytology has developed as a separate discipline from exfoliative cytology, but both are important parts of the total discipline of clinical cytology. In contrast to exfoliative cytology it cannot be used for screening, but is used in diagnosis of palpable lesions or lesions of internal organs which can be visualized by radiological techniques. Although the principles of cytological diagnosis remain the same, aspirated cells can present different artefacts, and material can be obtained from lesions in organs which do not provide exfoliated material. In consequence newcomers to this field need to gain experience to give reliable reports, and this book, together with Part 1 (monographs in Clinical Cytology, Vol. **4**), will be an essential addition to the library. It will also be of value to those who already report aspiration biopsy material because of the wide range of lesions studied.

E. B. Butler